# Trauma experience of youngsters and Teens: A key issue in suicidal behavior among victims of bullying?

**DOI:** 10.12669/pjms.301.4072

**Published:** 2014

**Authors:** Farhat Shireen, Himani Janapana, Sanila Rehmatullah, Hoor Temuri, Fatima Azim

**Affiliations:** 1 FarhatShireen, M.D.M.P.H, MentalHealthResearcher, BrookdaleHospitalandMedicalCenter Brooklyn,NewYork,USA.; 2 Himani Janapana, MD, Director of Clinical Education, Brookdale Hospital and Medical Center Brooklyn, NewYork, USA.; 3 Sanila Rehmatullah, MD, Residency Program Director, Department of Psychiatry. Brookdale Hospital and Medical Center Brooklyn, NewYork, USA.; 4 Ms. Hoor Temuri, BHS Brentwood TN; 5 FatimaAzim,MD, Consultant Psychiatrist, VA Veterans North Texas Health Care System, Dallas Texas,USA.

**Keywords:** School bullying, Suicide, Teens

## Abstract

This study examines the association between suicide and bullying among teenagers and adolescents in school besides exploring strategies to prevent acts of bullying. “Bullying” is a risk factor that is linked to suicidal ideation and attempts among school-age children and teenagers. Since youth suicide is an urgent and serious problem, we conducted a systematic review of 28 previous studies conducted in children and adolescents which examined the connection between bullying experiences and suicide.

***Data Collection:*** A literature search was carried out using 4 databases, without date description including: PubMed, PsychInfo, Medline and Google Scholar. The search terms contained: bullying, suicide and bullying, suicide in teens, school bullying, and peer victimization. An initial search generated about 97 articles; however, only 28 articles were appropriate for inclusion in the current review. Inclusion criteria was (1) Cross-sectional studies published from 1997-2013. (2) Study based on school bullying and suicidal risk in adolescents and teens 18 years or less (3) Studies had enough information to calculate effect sizes that did include a control group. (4) Studies based on gender discrimination. Papers that focused on specific populations, that did not include quantitative data, that did not use a control group of non-bullied subjects and studies based on cyber bullying, studies with longitudinal design were excluded.

The risk of suicide attempts was higher in girls, who were involved in bullying, either as the victim or perpetrator, than in boys. Depression, feelings of hopelessness and loneliness can develop in the child after being bullied for long periods of time; these feelings are indirectly related to suicidal ideation and attempts. Involvement in bullying increases the likelihood of suicidal ideation and attempts in children and teenagers.

## INTRODUCTION

Suicide is the third leading cause of death for people aged 15-24 in the USA.^1^ Teen suicide is not only a tragic global public health problem affecting young people. The surviving family members and friends live emotionally devastated lives after losing their loved one. Approximately 1 in 6 high school students has seriously considered suicide, and 1 in 12 has attempted it.^1^ Moreover, suicide rates among teens have been increasing for the last couple of years; from 6.3% in 2009, to 7.8% in 2011; and more youth suicides have been reported as a result of bullying.^2^ Furthermore, evidence indicates a strong association between bullying and suicide, as suggested by recent bullying-related suicide deaths.^1^

**School Bullying**

School bullying is a type of bullying that occurs in connection with education, whether inside or outside of school. Bullying can be physical, verbal, or social and is usually repeated over a period of time. Physical bullying includes hitting, kicking, and beating up, pushing, spitting, property damage, and/or theft. Verbal bullying includes teasing, mocking, name-calling, verbal humiliation/intimidation, threats, cruelty, extortion, and/or racist, sexist or homophobic taunts. Social bullying includes gossip, rumor spreading, embarrassment, alienation or exclusion from the group, and using the Internet, email or text messaging to threaten.^1^ Studies demonstrate that bullies are aggressive children.^1^ They see violence as the right way to interact with other children.^2^ They are insecure youth who believe that other children will harm them, so they fight to defend themselves, and also try to show that they’re strong. A lot of bullies are characterized by impulsive behavior.^1^^, ^^2^^.^

**Data Associated with Bullying in School**

Some of the effect of bullying that have been observed are: (1) It is estimated that 160,000 children skip school every day because of fear of attack or intimidation by other students (2) Ninety percent of 4th through 8th graders report being victims of bullying. (3) 1 in 7 students in Grades K-12 is either a bully or a victim of bullying.^1^Bullying statistics show that revenge is the strongest motivation for school shootings, and that harassment and bullying have been linked to 75% of school-shooting incidents.^2^Eighty-seven percent of students said shootings are motivated by a desire to “get back at those who have hurt them”.^2^

## DISCUSSION

According to the CDC, a national youth risk behavior survey showed that more than 1,700 adolescents aged 15-19 completed suicides each year.^2^^,^^3^ In 2009, the percentage of high school students who attempted suicide one or more times during the 12-month period was 6.3%, of which 8.1% were female and 4.6% were male.^1^^,^^3^ According to studies by Yale University, victims of bullying are between 2 to 9 times more likely to consider suicide than non-victims.^1^ Suicide is the third leading cause of death for people aged 15-24in the United States.^1^ A semi-annual survey on youth risk behavior by the Centers for Disease Control (CDC) in 2012 uncovered that approximately 1 in 6 high school students has seriously considered suicide, and 1 in 12 has attempted it.^2^ Furthermore, evidence indicates a strong association between bullying and suicide, as suggested by recent bullying-related suicide deaths.^1^ The more frequently a child is bullied, the more overwhelming it becomes. When a child becomes depressed, feelings of loneliness and sadness overcome them. They lose interest in activities, they blame themselves for every bad thing that happens around them, and they think life is not worth living. Some youth express their depression through violence and aggression, and at this point, they can become vulnerable to suicidal or destructive thoughts. As a result, most adolescent suicide attempts are caused by interpersonal conflicts. The intent of the behavior appears to be to effect change in the behaviors or attitudes of others. Therefore, the most important thing for the youth when he/she becomes suicidal is to get professional help immediately, because the suicidal feelings can be very robust.

**Table-I:** Different studies on suicide in adolescents

*Study Country*	*Population Studied/age group*	*Study Country*	*Population Studied/age group*
1-Rivers I, 2010. UK	2,002, 12 to 16 years boys & girls	16-Kim YS, 2009, Korea	6,043, 4 and 10 years boys & girls
2-Barker ED, 2008. UK	3,932, 14 to 16 years boys & girls	17-Park HS, 2006, Korea	1300 high school boys &girls
3-Wolke D, 2001, UK	2377 children 6-8 years boys& girls	18-Kim YS, 2005, Korea	1718, 7 and 8 grade, boys & girls.
4-Arseneault L, 2006, UK	2232 children, 5 and 7 years boys & girls	19-Hepburn L,2012,USA	1,838 youth in 9th-12th grade, boys & girls.
5-Rivers I, 2013, North England	1,009, high school boys & girls	20-Klomek AB, 2011, USA	96, 13 through 18 years boys & girls.
6-Luukkonen AH, 2009, Finland	508 adolescents 12-17 years boys & girls	21-Klomek AB, 2007 USA	2342, 9^_^12^th^ grade boys & girls.
7-Brunner R, 2007,Germany	5759, 9-grade, boys & girls.	22-Patrick DL, 2013, USA	27,752, 8, 10, and 12 grades boys & girls
8-Baldry AC, 2003, Italy	998 adolescents, 8 to 13 years boys & girls	23-Bauman S, 2013 USA	1491 high school students, boys & girls.
9-Rigby K, 1999, South Australia	845 secondary school, 12-16 boys & girls	24-Abdirahman, H.A Caribbean 2012	6780, middle school age, boys and girls.
10-Shaikh A M, 2013. Pakistan	4676, class 8-10, 14-16 boys & girls	25-LeVasseur MT, 2009 USA	2009 Youth survey, boys & girls
11-McMahon EM, Ireland 2012	1870, adolescents boys only	26-Hanley AJ, 2011 USA	448, 4-5 grade, 10-12 years boys & girls
12-Emmanuel R, 2007, Uganda	Uganda Global School-Based Health Survey, adolescent	27-Klomek AB 2008, Finland	2348 boys born in 1981.
13-Skapinakis P, 2011 Greece	5614, 16-18 years boys & girls	28-Van der, 2003, Netherlands.	4811 children, aged 9 to 13 boys & girls
14-Owusu A, west Africa 2011	7137 student high school		
15-Cui S, 2011, China,	8778, adolescents boys & girls		

**Link between Bullying and Suicide attempt and ideation in Cross-sectional studies**

***Bullying and risk of self-harm***

These cross-sectional studies have determined that victims of bullying express high levels of suicidal ideation, and the risk of attempted suicide is high as compared to non-victims.^4^^-^^6^ The association of being bullying to suicidal ideation/ suicide attempts is not only limited to students who were bullied but also reported by the bullies.^5^^,^^6^ These children have experienced rejection and are not popular among their peers, and develop early personality problems.^7^ Suicidal ideation and behavior may represent an attempt at reducing intolerable emotional states among these children and adolescents.^8^ However students who were both victim and perpetrators of bullying were at highest risk for both suicidal ideation and suicide attempt^4^^-^^11^ as compared with adolescents who were bullied but not involved in bullying behavior. One study reported that children who have multiple roles in bullying behavior such as, victim, bully, and bystander (Children who witnessed bullying, but they did not report it. These children may have feelings of discomfort about their behavior) were significantly more likely to report having had thoughts of ending their life.^6^

***Bullying affects on mental health***

Studies indicated that bullys/victims are at risk for suicidal ideation because of increased risk of mental health problems.^12^^-^^15^ These risks may be attributable to the development of intensified emotional arousal and poor impulse control.^16^^,^^17^ Findings also demonstrated the relationship between suicidal behaviors, bullying victimization experiences and depression which facilitate the association between bullying/victimization and suicide attempts.^18^ A recent study conducted in Pakistan revealed the effects of sleep patterns in bullied victims and how it further leads them towards serious considerations of attempting suicide.^19^ The study also discovered that children who are lonely and have insomnia were twice as likely to report suicidal ideation. In contrast to bullied children who do not have insomnia or feel lonely, these children were bullied within the past thirty days. Age and sex were not found to be statistically significant.^19^ Over time, victimization increased the probability of involvement in bullying as compared to bullying increased the likelihood of victimization.^10^ When symptoms of depression were controlled, suicidal ideation occurred most often among adolescents who were bullies.^10^

The intent of the behavior appears to be to effect change in the behaviors or attitudes of others. Therefore, the most important thing for the youth when he/she becomes suicidal is to get professional help immediately, because the suicidal feelings can be very robust. H. A. Abdirahman and colleagues in Caribbean studies found a strong association between bullying and poor mental health. They highlight the urgent need to develop useful approaches for reducing bullying among children and adolescents.^20^ Brunner and colleagues reported an indirect effect of bullying towards suicide. Their data suggest that there is a link present between bullying and deliberate self-harm (DSH). If the DSH behavior continues a strong association develops between DSH and suicidal behavior.^21^ Bullying has both direct and indirect effects on the suicide ideation and attempts. For example, a very important negative outcome of victimization may be increased risk of suicide attempt and death due to increasing feeling of hopelessness in the child.^22^ Evidence showed that verbal victimization was associated with an increased risk of developing hopelessness in elementary school children.^22^ Furthermore, studies revealed that problems in peer relationships are positively associated with suicide ideation and attempts, and that feelings of loneliness would increase the association.^4^^,^^23^ Evidence also showed that helplessness is significantly linked with possible suicide ideation among youth who observe bullying at school.^24^

**Dose-response relation**

Various researchers described a dose-response relation^1^^3^between victimization and suicidal ideation and attempt. The more frequent the exposure to bullying the higher the risk of the child developing these thoughts and ideation.^13^

**Bullying and Gender**

Various studies have reported association between victims of bullying and suicidal ideation and suicidal attempt when age, race/ethnicity, and gender were controlled for. Another aspect that was uncovered is the link between gender and school bullying as a risk for suicidal ideation. For example, it has been found that while the prevalence of suicidal ideation may be different. When an Irish study by McMahon et al examined the prevalence of bullying in Irish adolescent boys, they found that the odds ratio of lifetime self-harm was four times higher for boys who had been bullied than boys without this experience.^25^ Among girls, being bullied or bullying others are both potential impulsive risk factors for suicidal behavior. After adjusting for age, school factors, family factors and psychiatric disorders, there was a higher risk of suicide attempts in girls who were victims of bullying (OR=2.07, CI=1.04-4.11, p=0.037) or who bullied others (OR=3.27, CI=1.08-9.95, p=0.037).^26^

However, various studies find different effect of gender. A high prevalence of victimization was reported in an Italian school survey, and their regressions model showed the importance of child gender and age. They found that older girls were more at risk of suicidal ideation than either younger girls or boys.^12^ Barker and fellows further mentioned that the highest prevalence of bullies and bullying-victims was found among mid age adolescents. These youth have high/increasing bullying and high/increasing victimization trajectories.^10^ One study has found that older adolescents have a higher risk for exhibiting suicidal ideation. ^27^

Similarly, Kim and Luukkonena et al. found that females who were involved with school bullying as victim or perpetrator were at greater risk for suicide.^5^^,^^26^ Van der Wal and Park et al.^28^^,^^29^ described that girls have a strong association between being bullied and suicidal ideation.^29^ Similarly, depression facilitated the association between bullying/victimization and suicide attempts, but differently for males and females. A few studies showed that depression mediated the link between traditional bullying and suicide attempts for female only.^18^ Van der et al. discovered that the experience of being bullied has common outcomes of suicidal ideation among both boys and girls. These associations were greater for indirect than direct bullying. They further clarified that direct bullying had a substantial influence on depression and suicidal ideation in girls, but not in boys.^28^ Conversely, Rigby and Slee stated that the connection between being a bully and suicidal ideation applied to boy only.^30^

**Bullying and Sexual preferences**

Patrick and his colleague analyzed data from the 2010 Washington State Healthy Youth Survey collected in public school grades 8, 10, and 12 and their results found that children were being bullied more due to their sexual preferences.^31^ They discovered that 29% of male students and 27% of female students in these three grades were reported being bullied because of personal sexual preferences.^31^^,^^32^ The survey also revealed lower quality of life (QOL) scores associated with increased odds of depressed mood or consideration of suicide.^26^^,^^31^ By the same token another secondary data analysis of the 2009 New York City Youth Risk Behavior Survey used logistic regression to study the link between sexual identity, gender, ethnicity, bullying and suicide attempts. The outcome of bullying on suicide attempt was greatest among non-Hispanic sexual minority male youths (odds ratio = 21.39 vs. 1.65-3.38).^31^

Future long-term, prospective studies are needed to elucidate the causality between bullying and suicide as wells as the differential effects of gender on the association. Media plays a very significant role in covering and broadcasting suicide related teenage deaths. For example, if teenage suicide gets under reported, for good reason, there will be less risk of glorifying the behavior and less risk of clusters of suicidal behavior. Another positive media contribution can be to educate the public and to point out the seriousness of bullying and its related consequences.

School bullying is a major public health problem that demands the thoughtful attention of school systems, teachers, health care providers, policy makers, and families. School systems can collaborate with teachers, parents, students and the community to deal with bullying problems in their school, and come up with ways to respond to it effectively.

## SUGGESTIONS

1. One of the most effective suicide prevention strategies for youth is to provide awareness and educate students, parents, school officials, community members and peers on how to identify suicidal signs. This includes teaching them how to effectively respond to suicidal behavior, and how to provide support.
2. It is important to provide a safe environment in school and at home. To ensure this safety, parents can encourage children to openly discuss their school-related problems, including their peer relationships.
3. Studies have showed that 50-75% of children and youth who have been bullied have not informed their school. Some have told one of the parents, yet many remain silent. Therefore addressing bullying with frequent anti-bullying campaigns is likely to result in a substantial reduction in the suicide rate among students.

## Notes

***Acknowledgement:*** The authors would like to thanks to Ms. Deborah Dwoskin, Dr. Pierre Jean Noel and Dr. Jason Hershberger for their help and assistance in preparing this manuscript.
